# Interpersonal Similarity between Body Movements in Face-To-Face Communication in Daily Life

**DOI:** 10.1371/journal.pone.0102019

**Published:** 2014-07-11

**Authors:** Naoki Higo, Ken-ichiro Ogawa, Juichi Minemura, Bujie Xu, Takayuki Nozawa, Taiki Ogata, Koji Ara, Kazuo Yano, Yoshihiro Miyake

**Affiliations:** 1 Department of Computational Intelligence and Systems Science, Tokyo Institute of Technology, Yokohama, Kanagawa, Japan; 2 Institute of Development, Aging and Cancer, Tohoku University, Sendai, Japan; 3 Reseach into Artifacts, Center for Engineering, the University of Tokyo, Kashiwa, Japan; 4 Center Research Laboratory, Hitachi Ltd., Tokyo, Japan; Centre de Physique Théorique, France

## Abstract

Individuals are embedded in social networks in which they communicate with others in their daily lives. Because smooth face-to-face communication is the key to maintaining these networks, measuring the smoothness of such communication is an important issue. One indicator of smoothness is the similarity of the body movements of the two individuals concerned. A typical example noted in experimental environments is the interpersonal synchronization of body movements such as nods and gestures during smooth face-to-face communication. It should therefore be possible to estimate quantitatively the smoothness of face-to-face communication in social networks through measurement of the synchronization of body movements. However, this is difficult because social networks, which differ from disciplined experimental environments, are open environments for the face-to-face communication between two individuals. In such open environments, their body movements become complicated by various external factors and may follow unstable and nonuniform patterns. Nevertheless, we consider there to be some interaction during face-to-face communication that leads to the interpersonal synchronization of body movements, which can be seen through the interpersonal similarity of body movements. The present study aims to clarify such interaction in terms of body movements during daily face-to-face communication in real organizations of more than 100 people. We analyzed data on the frequency of body movement for each individual during face-to-face communication, as measured by a wearable sensor, and evaluated the degree of interpersonal similarity of body movements between two individuals as their frequency difference. Furthermore, we generated uncorrelated data by resampling the data gathered and compared these two data sets statistically to distinguish the effects of actual face-to-face communication from those of the activities accompanying the communication. Our results confirm an interpersonal similarity of body movements between two individuals in face-to-face communication, for all the organizations studied, and suggest that some body interaction is behind this similarity.

## Introduction

Individuals are embedded in social networks in which they communicate with others in their daily lives. Communication is an exchange of messages between individuals, in which verbal and nonverbal messages are transmitted through bodily movements. Individuals can often communicate with others without realizing it. So-called “smooth communication” is considered to include communication at an unconscious level. Because smooth communication is the key to maintaining social networks, measuring the smoothness of face-to-face communication is an important issue.

We can use the similarity of the body movements between two individuals communicating with each other as an indicator of the smoothness of the communication. There are many studies on synchrony phenomena in body movements, which seem to reflect unconscious communication. Studies investigating such synchrony phenomena during face-to-face communication have been conducted in the field of psychology [1]–[13]. For instance, it has been observed that the voice of the speaker is in synchronization with the body movement of the hearer in communication between a mother and an infant and between adults [1], [2]. This phenomenon is a well-known example of body movement synchronization that arises unconsciously. It has been reported that, when solving a puzzle, the puzzle solver's upper body posture is synchronized with that of the puzzle giver [3]. It has also been found that, when two individuals tell each other jokes, their body movements are synchronized [4]. In addition, many studies have investigated the effects of upper body synchronization. For instance, one study showed that if an interviewer nods in response to an interviewee's remarks, the interviewee seems to talk more and to become more confident [5]. Another study showed that when two individuals have a conversation, they nod in synchronization when a context is shared [6]. Although the causality of the relationship between the synchronization of nods and the sharing of contexts has yet to be revealed, other studies suggest that the synchronization of body movement is based on smooth communication through shared contexts [7]–[13]. In this way, the synchronization of body movements that include nodding has been found, to a greater or lesser extent, in a variety of cultures. Furthermore, synchronization can be well defined in mathematical terms. It should therefore be possible to estimate the smoothness of daily face-to-face communication in social networks quantitatively, through studying the synchronization of body movements such as nodding.

However, there is a serious problem here. The above studies reported findings in experimental environments, rather than in daily life. In experimental environments, conditions such as the communication partner and time are decided in advance [3], [4], [13]. This is in contrast to daily life, where we cannot decide beforehand when communication will start or how long it will continue. It is known that the duration of real face-to-face communication can vary from one minute to one hour, following a power-law distribution [14]. Moreover, it is unpredictable where or with whom we will be communicating. Therefore, because so many factors are intricately intertwined in communication in daily life, compared with controlled experimental environments, the body movements of two individuals during routine face-to-face communication will follow unstable and nonuniform patterns. In addition, influences from other people present in the social network can affect an individual's body movement through ways other than direct face-to-face communication such as conversation. Such factors can lead two individuals to synchronize their movements simply by seeing each other face to face, even without direct conversation. Therefore, it is quite difficult to identify the interpersonal synchronization of body movements in open environments, given the effects of such social networking. Nevertheless, we consider there to be some interaction during face-to-face communication that leads to the interpersonal synchronization of body movements, which can be seen through the interpersonal similarity of body movements [15], [16].

The purpose of the present study, therefore, is to demonstrate the presence of such interaction between body movements during face-to-face communication in daily life. We focused on daily face-to-face activities in actual organizations of more than 100 people for a period of between one and two months. First, we obtained data on the frequency of upper body movements including nods and other gestures for each individual during face-to-face communication. The data was measured by the World Signal Center (Hitachi Ltd., Tokyo, Japan), using a badge-type wearable sensor developed for network analysis. Next, we evaluated the interpersonal similarity of body movements from the data gathered, using the measured differences between the body movement frequencies for each individual. In addition, we generated uncorrelated data by resampling these data and compared these two data sets statistically, in order to distinguish the effects of actual face-to-face communication, including temporal structures, from those caused by other activities.

## Methods

### Subjects for Analysis

Table 1 gives information about the organizations in our analysis, namely the type of organization, the number of subjects, and the number of measurement days. Our analysis was performed using extensive data from seven organizations (A to G) recorded over a period of between one and two months. Each organization was a department of a company and employed more than 100 people. The number of subjects is the number of participants who wore a measuring device for at least one day during the measurement period.

**Table 1:** Organizations for analysis.

Organization	Type	Subjects	Days
A	Consultant	134	33
B	Research & Product Development	163	47
C	Wholesale	211	48
D	Product Development Support	219	59
E	Product Development	144	64
F	Product Development	109	59
G	Product Development	124	61

### Measuring Device

In the present study, we focused on those oscillating movements of the upper body, such as nods and other gestures, which occur during everyday conversation. These oscillating movements were captured by an acceleration sensor similar to that used recently in the measurement of body movements [17]. We obtained data on the body movements involving pairs of subjects during face-to-face communication in the organizations A to G shown in Table 1. The data was measured and collected by the World Signal Center using a badge-type wearable sensor called the Hitachi Business Microscope (Hitachi High-Tech Science Corporation, Tokyo, Japan). The data acquisition was approved by the administration division of Hitachi Ltd. Informed consent in writing was obtained for each subject. The collected data are available to anyone by making an application for membership in the World Signal Center [18].

**Figure 1:**
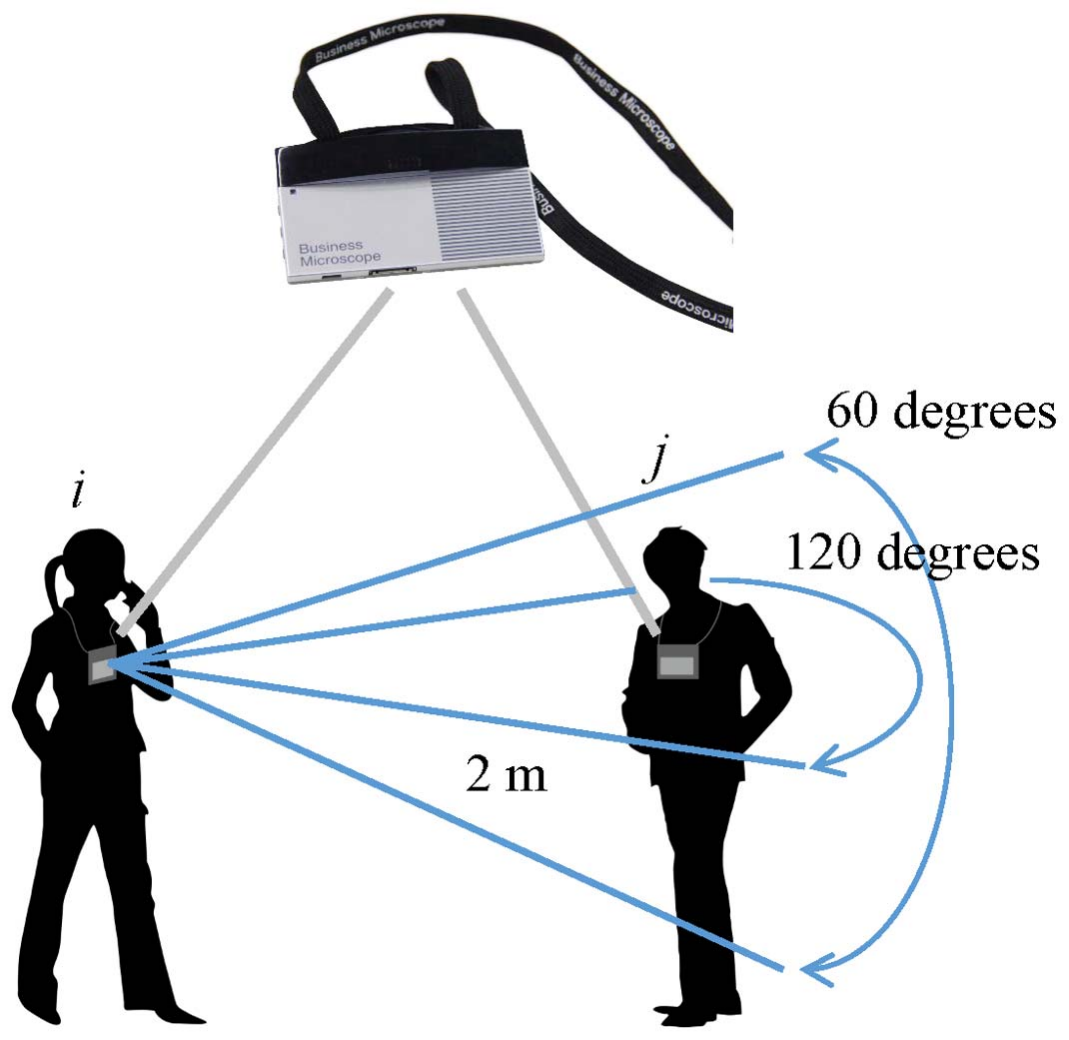
Wearable device (Hitachi Business Microscope). The device is attached to the upper torso of the subject. The device has a three-axis acceleration sensor and an infrared sensor. The acceleration sensor detects accelerations in upper torso movement. The infrared sensor detects face-to-face contact events. The subject puts on this device when arriving at work and takes it off when leaving.

The measuring device was equipped with an acceleration sensor [19], [20]. As shown in Figure 1, the wearable sensor was attached to the upper torso of each subject on arrival at work and removed when leaving work, over a period of between one and two months. Each device recorded time series data on the acceleration of the subject's body (upper torso) along each spatial axis throughout the entire period of measurement. The device was further equipped with an infrared sensor. This enabled the subjects involved in each face-to-face communication to be identified within the range of infrared communication (a horizontal angle of 120 degrees, a vertical angle of 60 degrees, and a radius of 2 meters). To measure face-to-face time, the data were organized into one-minute blocks of time, such that the device recorded a score of 1 block if a face-to-face contact event was detected at least once during the one-minute period.

### Body Movement

From the acceleration data measured in the above manner, the frequency of body movements for each one-minute block was calculated as follows. The wearable sensor acquired time series data on the acceleration of the upper torso of each subject for each spatial axis at 50 Hz, enabling time series data for an acceleration norm to be calculated. Next, the frequencies *n_i1_*(*t_k_*)−*n_i6_(t_k_)* Hz of subject *i* for 10 seconds every minute *t_k_* was calculated. This calculation was performed by counting the number of times that the time series data of the acceleration norm crossed a reference line that was set as the average value of the time series data of the acceleration norm every 10 seconds. Finally, the average frequency *ω_i_*(*t_k_*) Hz by the minute, as a measure of the frequency of body movement for subject *i*, was calculated as follows:
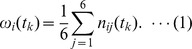



We obtained the frequency data calculated as above from the World Signal Center.

### Face-To-Face Contact Events

In addition, as described above, if a face-to-face contact event between subjects *i* and *j* was detected by the wearable sensor within the range of infrared communication at least once during a one-minute block, the wearable device recorded a score of “1” for that block. Note that when subjects *i* and *j* communicated with subject *h* at time *t_k_*, even if the distance between subjects *i* and *j* was more than two meters, a score was recorded. This is a form of triangle closure used as a condition for considering group communication. For instance, consider the case of many subjects gathering in a conference room for a discussion. In such a situation, there will often be a pair of subjects more than two meters from each other during face-to-face communication. Because this situation is frequently seen in real organizations, we considered the effect of group communication in our analysis.

### Interpersonal Similarity in Body Movements

Then, the problem is to select a suitable physical quantity by which to evaluate the interpersonal similarity between the body movements of two subjects during face-to-face communication. In general, oscillation phenomena have waveforms characterized by frequency, phase, and amplitude. In particular, the entrainment or synchronization of nonlinear oscillators is well represented by phase difference [21]. However, because time series data on the body movement of a subject in a social network is not stable, it is difficult to evaluate interpersonal similarity between body movements from the phase difference. In the present study, therefore, we evaluated a frequency difference to capture the interpersonal similarity:
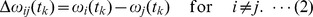



Here, *ω_i_*(*t_k_*) and *ω_j_*(*t_k_*) denote the body movement frequencies of subjects *i* and *j* at time *t_k_*, respectively. As the frequency difference Δ*ω_ij_*(*t_k_*) approaches 0 during face-to-face communication, the degree of interpersonal similarity between the subjects' body movements increases. In this study, the frequency difference Δ*ω_ij_*(*t_k_*) was not calculated for situations when *ω_i_*(*t_k_*) = 0 or *ω_j_*(*t_k_*) = 0. Note that, when subjects *i* and *j* communicated with subject *h* at time *t_k_*, even if the distance between subjects *i* and *j* was more than two meters, Δ*ω_ij_*(*t_k_*) was calculated to capture their interpersonal similarity.

### Statistical Analysis

As described above, we calculated frequency difference Δ*ω_ij_*(*t_k_*) for all pairs *i* and *j* in each organization who communicated face to face at any time during the measurement period. Next, we constructed data set *Y* (the “original data set”) with all the calculated values as elements:
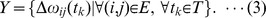



Here, *E* denotes the label set that shows subjects *i* and *j* in face-to-face communication, and *T* is the set of times when they are communicating. We conducted a statistical analysis of the distribution of frequency differences Δ*ω_ij_*(*t_k_*) in the original data set *Y* to investigate interpersonal similarity in body movements for face-to-face communication pairs in the organizations A to G described above. In the analysis, to estimate the interpersonal similarity of face-to-face communication reliably, we eliminated the one-minute blocks from the beginning and ending of periods of face-to-face communication measured continuously in original data set *Y* because of the insufficiency of the original data in these blocks. For instance, if face-to-face communication continued for five minutes, only the middle three minutes were considered in the analysis. This allowed for the possibility that subjects did not necessarily communicate with each other during the one-minute block at the beginning and ending of the communication. The reason is that the device could actually detect face-to-face contact events occurring only once during a one-minute period. The data selected as above were relevant for analysis in terms of the aim of the present study.

Furthermore, we need to identify the effects of body interaction between two subjects during face-to-face communication. It should be noted here that other factors can cause body movements, in addition to face-to-face communication such as conversation. For instance, it is reported that body movement frequencies during face-to-face communication depend on the type of communication, but do not vary much from subject to subject [22]. Therefore, consider the situation of subjects *i* and *j* being in an area where they are mutually detectable by their wearable sensors, but subject *i* is communicating with subject *m* and subject *j* is communicating with subject *n*. In this situation, although subject *i* does not communicate with subject *j* directly, subjects *i* and *j* may exhibit body movements based on their communications with subjects *m* and *n*, respectively. In this case, therefore, *ω_i_*(*t_k_*) may be almost identical to *ω_j_*(*t_k_*). This is also true when individuals *i* and *j* are not mutually within the range of infrared communication. As a result, although subjects *i* and *j* are not actually in face-to-face communication, there may be an apparent reduction in frequency difference Δ*ω_ij_*. It is therefore insufficient merely to compare face-to-face contact with noncontact events. We need to identify the effects of actual face-to-face communication including the temporal structure from the original data set *Y*. To distinguish body movements caused by face-to-face communication from ones caused by these other factors, we constructed the resampled data set Y^*^ (the “resampled data set”) from the original data set *Y*:
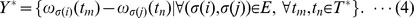



Here, *σ* is an operator that rearranges each component randomly and T^*^ is a set of times randomly selected from the elements of set *T*. However, the resampled data set Y^*^ does not include the original data set *Y*.

We then compared the distribution of the original data set *Y* (the “original distribution”) with the distribution of the resampled data set Y^*^(the “resampled distribution”). In particular, we calculated the standard deviation *θ^SD^* and kurtosis *θ^kurt^* of the distributions. This was to investigate the spread of each distribution and the deviation of the distribution from the center (Δ*ω* = 0). For instance, for the original distribution, these were calculated as follows:
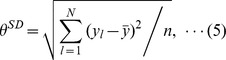





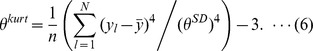



Here, *y_l_* is an element of original data set *Y*, *N* is the number of the elements, and 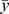 is the average value. From equation (2), it is clear that 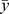 = 0 in this case. The kurtosis *θ^kurt^* is defined as the difference from the kurtosis of the Gaussian distribution, rather than the kurtosis of the original data set *Y* itself.

## Results

### Body Movement Frequency

**Figure 2:**
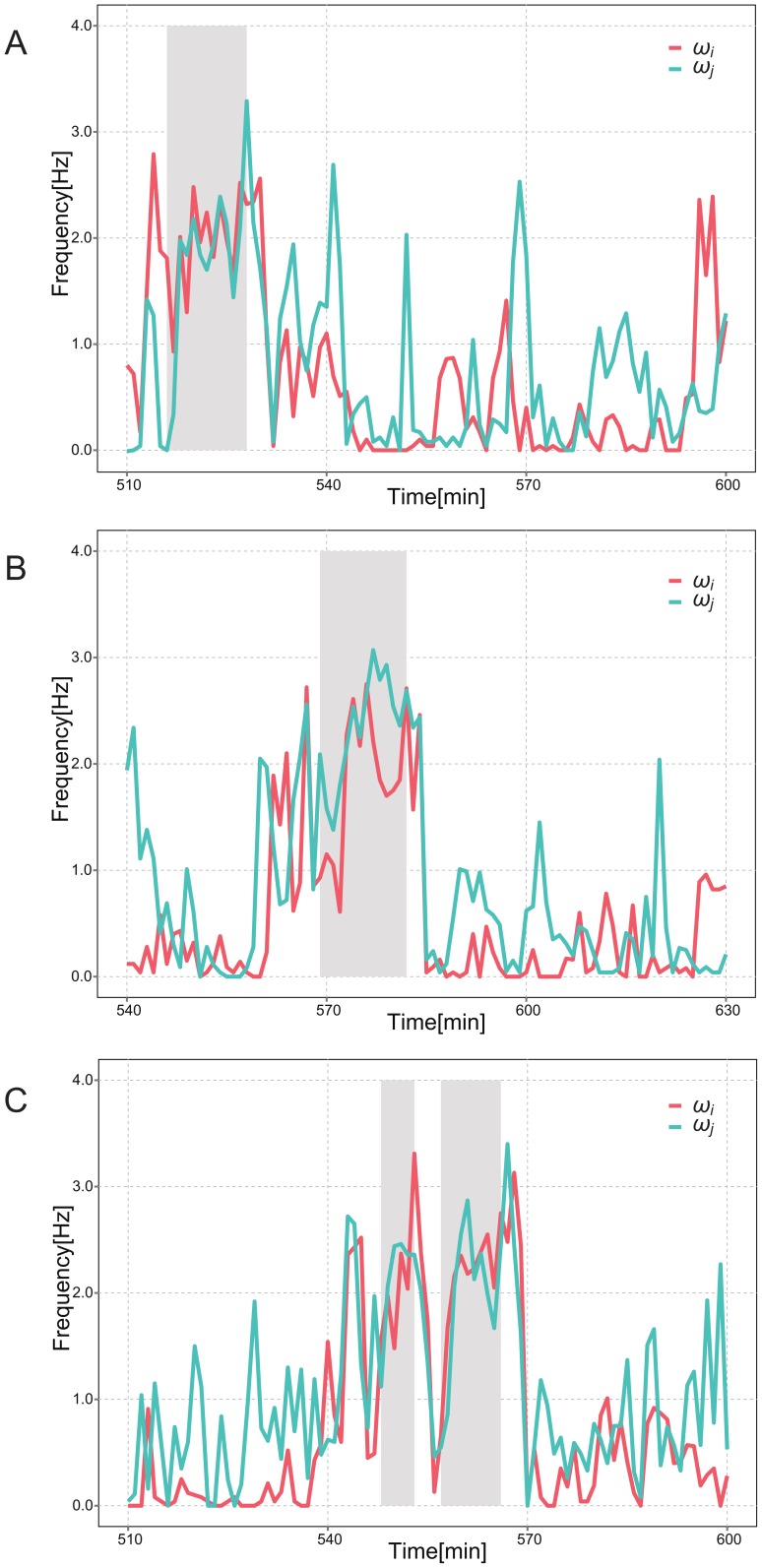
Time series data for the frequency of body movements. (A) to (C) show the body movement frequencies of two subjects in organization D during the morning of one day. The gray regions denote face-to-face time for the two subjects.

As a specific example, Figures 2A to 2C each show the time series data for the body movement frequencies of two subjects with ID numbers 39 and 65 from organization D during the morning of one day. Figure 2A shows the data for the period 8:30 am to 10:00 am on day 53, Figure 2B shows the data for the period 9:00 am to 10:30 am on day 54, and Figure 2C shows the data for the period 8:30 am to 10:00 am on day 55. In these figures, the gray regions represent face-to-face contact time for the two subjects. Although Figures 2A to 2C show an example of the tendency to increase both body movement frequencies for two subjects during face-to-face communication, other cases are more complicated. That is, all the data do not necessarily show such a tendency. We therefore investigated the body movement frequencies of subjects during face-to-face communication.

**Figure 3:**
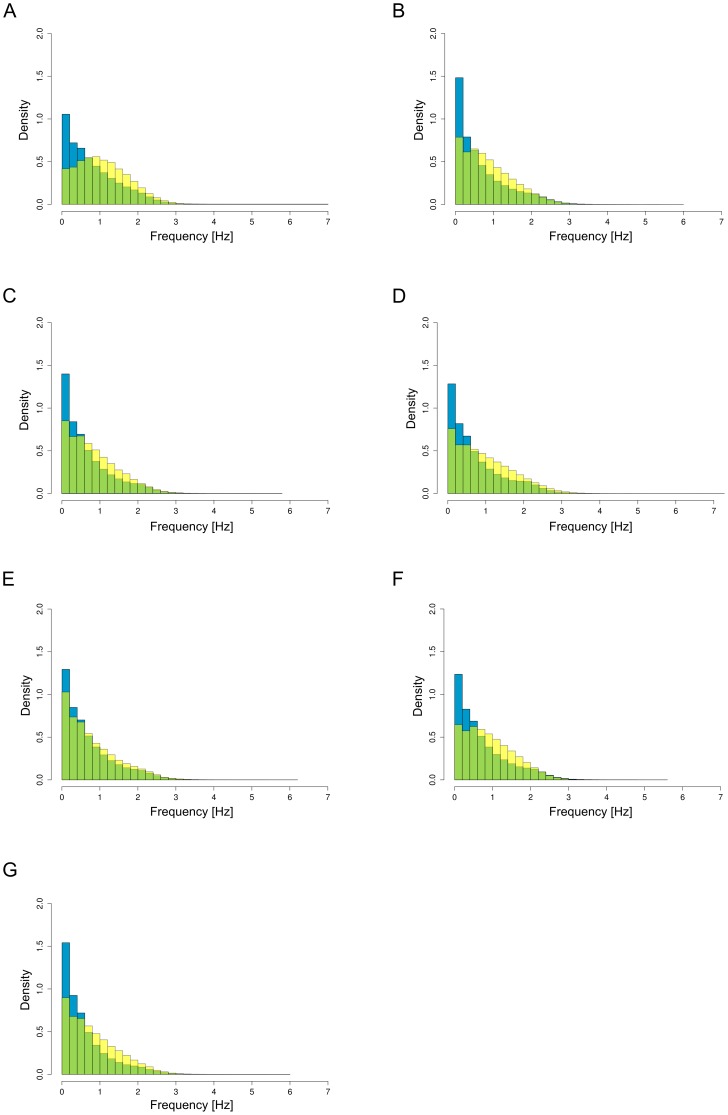
Frequency distribution of body movements. The distribution composed of the yellow and green histograms of (A) to (G) show the distributions of the body movement frequencies for each subject during face-to-face communication over the measurement period in organizations A to G, respectively. The distribution composed of the blue and green histograms of (A) to (G) show the distributions of the body movement frequencies for the same subjects during the period when they did not communicate with anyone over the measurement period.

Figures 3A to 3G show the distribution of the body movement frequencies of subjects under two different states. In these figures, the distribution composed of the yellow and green histograms (the “face-to-face communication distribution”) shows the distribution of the body movement frequencies for each subject during face-to-face communication over the measurement period for organizations A to G, respectively. The distribution composed of the blue and green histograms (the “non-communication distribution”) shows the distribution of the body movement frequencies for the same subjects during the period when they did not communicate with anyone over the measurement period. Here, the green histograms are the overlapped regions of the face-to-face communication and non-communication distributions. These figures indicate that the face-to-face communication distribution has a higher density than the non-communication distribution in the approximate range more than 0.6 Hz for all organizations A to G. The figures also indicate that the density difference shown as the yellow histograms has the peak in the approximate range of 1.0 to 1.4 Hz for organizations A to G. We further investigated typical statistical indicators of the two frequency distributions. Table 2 shows the mean and standard deviation of the two frequency distributions. This table indicates that the mean of the face-to-face communication distribution has a larger value than that of the non-communication distribution for all organizations A to G whereas the standard deviation has a subtle difference between the two frequency differences. This means that the body movement frequency of each subject shifts toward higher frequencies in the face-to-face communication distribution. This difference between the two frequency distributions leads to a further investigation of the body movements during face-to-face communication.

**Table 2:** Mean and standard deviation of the face-to-face communication and non-communication distributions.

	Mean		Standard deviation	
	Face-to-face communication	Non-communication	Face-to-face communication	Non-communication
A	1.082	0.796	0.647	0.653
B	0.875	0.698	0.643	0.684
C	0.839	0.668	0.634	0.629
D	0.977	0.739	0.731	0.686
E	0.805	0.702	0.647	0.650
F	0.929	0.735	0.637	0.672
G	0.841	0.607	0.652	0.601

### Interpersonal Similarity

Figures 3A to 3G also indicate that the body movement frequencies of each subject during face-to-face communication are distributed to a wide range. Furthermore, Figures 2A to 2C indicate that frequency difference Δ*ω_ij_*(*t_k_*) approaches 0 Hz during face-to-face communication. That is, there is a possibility that frequency difference Δ*ω_ij_*(*t_k_*) approaches 0 Hz during face-to-face communication in a wide frequency range. We therefore focused on the original distribution defined as the absolute frequency difference.

**Figure 4:**
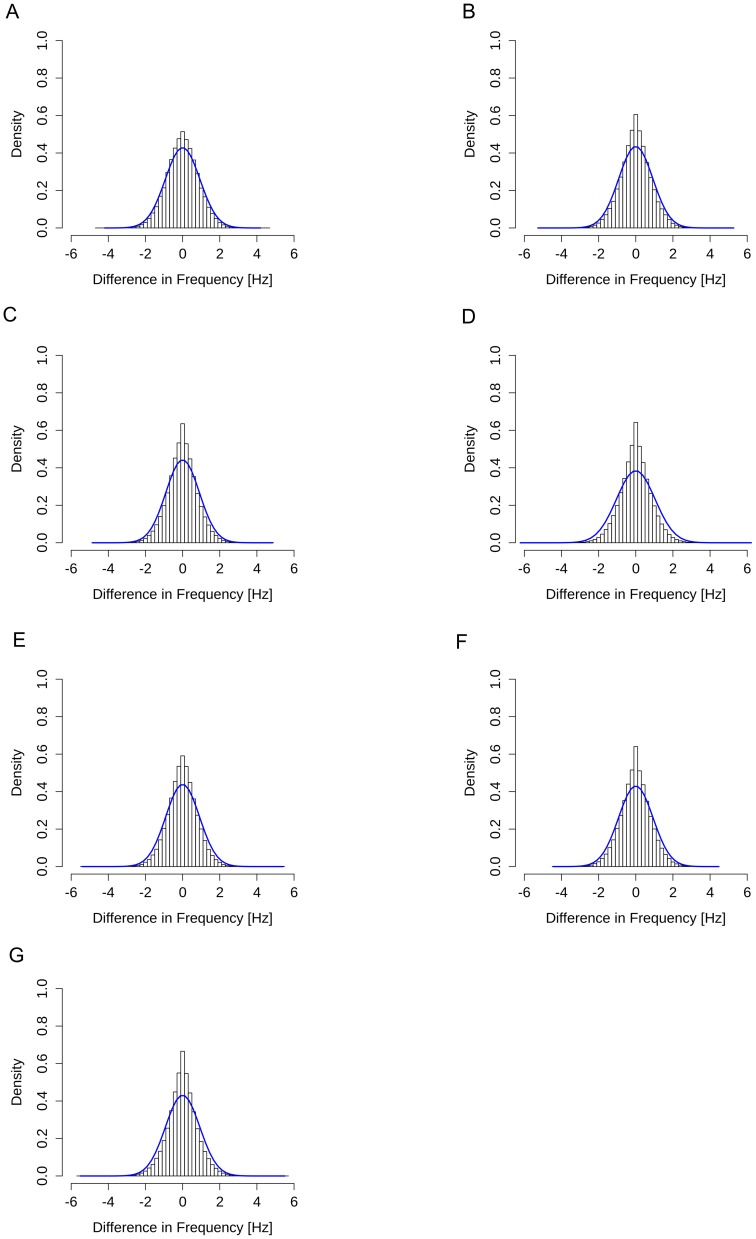
Original and resampled distributions of organizations A to G. (A) to (G) show the original and resampled distributions for organizations A to G, respectively. In each figure, the histogram represents the original distribution and the solid blue line represents the average resampled distribution.

Here, the original distribution was compared with the resampled distribution to investigate the effects of body interaction between two subjects during face-to-fare communication. Figures 4A to 4G show the original distribution for the organizations A to G and the average of the distributions obtained by resampling the original data set *Y* 500 times. In the figures, the histograms represent the original distributions and the solid lines represent the average resampled distributions. Table 3 shows the standard deviation *θ^SD^* and kurtosis *θ^kurt^* for the original distribution and the standard deviation θ^SD*^ and kurtosis θ^kurt*^ for the average resampled distribution. Figures 4A to 4G and Table 3 together suggest that, for all organizations A to G:

**Table 3:** Standard deviation and kurtosis of the original and resampled distributions.

	*θ^SD^*	*θ^SD^* * (SD*)	*θ^kurt^*	*θ^kurt^* * (SD*)
A	0.836	0.931 (2.21×10^−3^)	0.112	−0.130 (1.73×10^−2^)
B	0.796	0.914 (1.88×10^−3^)	0.369	0.067 (1.62×10^−2^)
C	0.779	0.902 (1.30×10^−3^)	0.593	0.149 (1.20×10^−2^)
D	0.812	1.037 (1.41×10^−3^)	0.607	0.130 (1.30×10^−2^)
E	0.767	0.913 (1.46×10^−3^)	0.549	0.100 (1.24×10^−2^)
F	0.791	0.928 (1.74×10^−3^)	0.430	-0.085 (1.43×10^−2^)
G	0.783	0.925 (2.08×10^−3^)	0.930	0.170 (1.78×10^−2^)

* SD denotes the standard deviation of θ^SD*^ and θ^kurt*^.

1. The standard deviation of the original distribution is significantly smaller than that of the average resampled distribution, and
2. The kurtosis of the original distribution is significantly larger than that of the average resampled distribution.

This means that the original distribution has a narrower spread than the average resampled distribution, whereas the original distribution has a greater concentration around the center than the average resampled distribution. In addition, the central limit theorem predicts that the average resampled distribution will approach the standard Gaussian distribution asymptotically as the number of resamplings increases. Therefore, it is expected that the original distribution will have a greater concentration around the center than the standard Gaussian distribution. In terms of equation (2), this suggests a positive correlation between the body movement frequencies of two subjects during face-to-face communication. It is therefore confirmed that there is a tendency for the body movements of two subjects in face-to-face communication to be similar for all organizations A to G.

## Discussion

In the present study, we have attempted to clarify whether the body movements of two individuals are similar during routine face-to-face communication in an open social network environment. First, we defined the body movement frequency for each individual in seven organizations using a wearable sensor. Second, we conducted a statistical evaluation of the interpersonal similarity of body movements between two individuals during face-to-face communication based on these data, using the difference between their movement frequencies. As a result, it was found that the body movements tend to be similar during face-to-face communication in all the organizations. From this result, we confirmed the interpersonal similarity between the body movements of pairs of individuals engaged in daily face-to-face communication in real social networks.

Table 3 shows that the original distribution has a greater concentration toward the center than does the standard Gaussian distribution, which is based on the uncorrelated data set *Y* ^*^ obtained by resampling the original data set *Y*. This result reflects some local body interaction between two individuals during face-to-face communication. One such type of local body interaction is known as mutual entrainment [23], [24]. In addition, this may reflect the global temporal structure of the original data set *Y* because, in our resampling method, both the pairs of face-to-face contact events and their time series were shuffled to secure adequate numbers of pairs for resampling. This suggests that global temporal variations in body interaction may be involved in the mechanism that generates interpersonal similarity between the body movements of two individuals during face-to-face communication in real organizations. To investigate this possibility, we would need to investigate further the global temporal structure of the face-to-face communications in each organization.

As shown in Figures 3A to 3G, the body movement frequencies found in the present study were distributed within the approximate range of 0 to 6 Hz. The body movement frequencies during face-to-face communication depend on the type of communication [22]. For instance, the frequency band of 0 to 1 Hz is liked to individual's behaviors such as Web browsing and listening, and the frequency band of 1 to 4 Hz is related to individual's behaviors such as talking, talking with dynamic gestures, and excited discussion. This indicates that daily face-to-face communication in social networks takes many varied forms. In addition, these figures indicate that the frequency range of about 0.6 to 2 Hz is a dominant region of the face-to-face communication distribution. We therefore plan to analyze the interpersonal similarity of body movements in terms of frequency ranges.

In the present study, we analyzed the upper body movement of the individuals only in terms of frequency. The phase of upper body movements, in addition to their frequency, is an important indicator for analyzing synchronization because it represents the relative timing of events during face-to-face communication. However, because the time series data on the body movement of an individual is not stable, it is difficult to evaluate interpersonal similarity between body movements from phase differences. As a first order of approximation, it was therefore reasonable for us to analyze the interpersonal similarity of body movements during face-to-face communication such as daily conversation in terms of frequency. However, we plan to analyze the interpersonal similarity of body movements in terms of phase differences. It will also be important to investigate how individuals control the timing of their body motions during face-to-face communication. For such an investigation, we would need to add phase difference information to the present analysis.

Our analysis also focused on the upper body movement of the individuals such as nodding during face-to-face communication. Upper body gestures such as nods are common phenomena in Japanese culture. However, we would have to analyze the movements of other parts of the body, such as hands or arms, in order to make sure of the dependence on the type of movement. If the interpersonal similarity of gestures during face-to-face communication results from some sort of body interaction, it would appear that the similarity could be explained by the mutual entrainment mechanism discussed above. In that case, we would expect to obtain the same results as in the present study for the movements of hands or arms during face-to-face communication.

In the present study, the data for face-to-face communication was acquired with predetermined sampling rates (1 minute for the detection of face-to-face contact events, and 10 seconds for the calculation of average acceleration). It would be very interesting to investigate the robustness (or universality) of the original distribution for these rates. However, the present study did not allow for such an investigation because we did not have access to the raw data for acceleration norms. We therefore have to seek other approaches for investigating the robustness of the distribution of body movement frequencies, such as using our own direct measurements of acceleration data.

By using the method of the present study, we can analyze social network structures from the viewpoint of the similarity of body movements during face-to-face communication in daily life. In general, social network structures change over time and space. In future work, therefore, we should analyze the body movements of two individuals during face-to-face communication in the context of such temporal and spatial information. Furthermore, the ways in which temporal and spatial changes in body movements may alter the social networks themselves remains an important issue.
